# 3D Reconstitution of the Neural Stem Cell Niche: Connecting the Dots

**DOI:** 10.3389/fbioe.2021.705470

**Published:** 2021-10-28

**Authors:** Konstantinos Ioannidis, Ioannis Angelopoulos, Georgios Gakis, Nikolaos Karantzelis, Georgios A. Spyroulias, Zoi Lygerou, Stavros Taraviras

**Affiliations:** ^1^ Department of Physiology, School of Medicine, University of Patras, Patras, Greece; ^2^ Department of Development and Regeneration, Prometheus Division of Skeletal Tissue Engineering, Skeletal Biology and Engineering Research Center, KU Leuven, Leuven, Belgium; ^3^ Department of Hematology, Oncology and Stem Cell Transplantation, University Medical Center Freiburg, Freiburg, Germany; ^4^ School of Pharmacy, University of Patras, Patras, Greece; ^5^ Department of General Biology, School of Medicine, University of Patras, Patras, Greece

**Keywords:** 3D organotypic models, organ on a chip (OCC), subventricular zone (SVZ), stem cell niche, neural stem cells (NSC)

## Abstract

Neural stem cells (NSCs) are important constituents of the nervous system, and they become constrained in two specific regions during adulthood: the subventricular zone (SVZ) and the subgranular zone (SGZ) of the dentate gyrus in the hippocampus. The SVZ niche is a limited-space zone where NSCs are situated and comprised of growth factors and extracellular matrix (ECM) components that shape the microenvironment of the niche. The interaction between ECM components and NSCs regulates the equilibrium between self-renewal and differentiation. To comprehend the niche physiology and how it controls NSC behavior, it is fundamental to develop *in vitro* models that resemble adequately the physiologic conditions present in the neural stem cell niche. These models can be developed from a variety of biomaterials, along with different biofabrication approaches that permit the organization of neural cells into tissue-like structures. This review intends to update the most recent information regarding the SVZ niche physiology and the diverse biofabrication approaches that have been used to develop suitable microenvironments *ex vivo* that mimic the NSC niche physiology.

## Introduction

### Adult Stem Cell Niche Development and Maturation

Rodent’s mammalian cortex neurogenesis begins with the generation of neuroepithelial stem cells (NESCs). This process is conversed between mammals and humans in the in the subventricular zone (SVZ) (Ernst and Frisén, 2015). NESCs undergo symmetric divisions in order to generate a pool of radial glial cells (RGCs) that will later generate nascent projection neurons (Cadwell et al., 2019). These neurons will then migrate from the ventricular zone to the cortical plate, where the earliest neurons form the preplate, which is then split into the marginal zone and subplate regions (Molnár et al., 2019). During the cerebral cortex development, a six-layered neocortex is generated, and its organization follows an inside-out pattern, with the earlier-migrating progenitors giving rise to the deeper layers of the cerebral cortex, and the later-migrating progenitors giving rise to the more superficial ones (Agirman et al., 2017). After embryonic neurogenesis is complete, the radial scaffold of the RGCs detaches from the apical surface (Molnár et al., 2019) and through asymmetric divisions gives rise to both astrocytes and ependymal cells. Neurogenesis, however, is not a process that stops in adulthood. Following the completion of cerebral cortex development, neural stem cells reside within a specialized microenvironment in the adult brain, called the neural stem cell (NSC) niche (Andreotti et al., 2019). So far, two main adult NSC niches have been described, namely, the ventricular–subventricular zone (V-SVZ) and the subgranular zone (SGZ) stem cell niche. The V-SVZ, which is the main point of interest in this mini-review, is the main region where new inter-neurons for the olfactory bulb are generated (Altmann et al., 2019). The SGZ, on the other hand, is the region where new hippocampal neurons are generated, a process which is thought to play a critical role in memory consolidation (Butti et al., 2014).

### SVZ Cell Populations and Functions

The main cell populations that comprise the SVZ stem cell niche are briefly reviewed below [for a detailed review refer to (Del Bigio, 1995)]. The mammalian SVZ is comprised of four layers (ependymal, hypocellular, astrocytic ribbon, and transitional layers) (Altmann et al., 2019) and, as described above, is the main region where the generation of new neural cells takes place. The NSCs found in this area, called B1 cells, exhibit astroglial characteristics and give rise to B2 cells, which also exhibit astroglial characteristics but lack an apical contact with the CSF (Obernier and Alvarez-Buylla, 2019). B1 cells also give rise to transient amplifying cells (IPCs or C cells), which subsequently differentiate toward young neurons (neuroblasts or A cells) (Obernier and Alvarez-Buylla, 2019). Ependymal cells, which are also derived from glial lineage but have epithelial characteristics, are cuboidal-to-columnar ciliated cells forming a thin sheet across the ventricles (Del Bigio, 1995) and are known to play a vital role in a variety of processes, including neural development as well as trophic and metabolic regulation of neural cells (Del Bigio, 2010) and cerebrospinal fluid (CSF) circulation (Spector et al., 2015). In addition, ependymal cells surround B1 cells by forming the so-called pinwheel structures (Paez-Gonzalez et al., 2011), which are crucial for proper regulation of adult neurogenesis. Specifically, a strong adhesion between ependymal and B1 cells is secured, allowing NSCs to contact both the CSF and the interstitially located blood vessels (Mirzadeh et al., 2008). Moreover, this strong adhesion between cells allows the process of neurogenesis to be influenced by adhesion-mediated and paracrine signaling, which has been analytically reviewed by Harkins et al. (2021). The crucial role that ependymal cells play, as mentioned above, is the control of proper CSF flow and the structural integrity maintenance of the SVZ stem cell niche (Paez-Gonzalez et al., 2011). In some cases, the disruption of the physiology of the ependymal cell function can lead to disorders of CSF dynamics, with the clinical entity of hydrocephalus constituting a typical paradigm (McAllister, 2012). Recent findings suggest that ependymal cells and adult neural stem cells share a common progenitor, and Geminin superfamily members control the process (Kyrousi et al., 2017).

### ECM Architecture and Physiological Cell Secreted Factors

**FIGURE 1:**
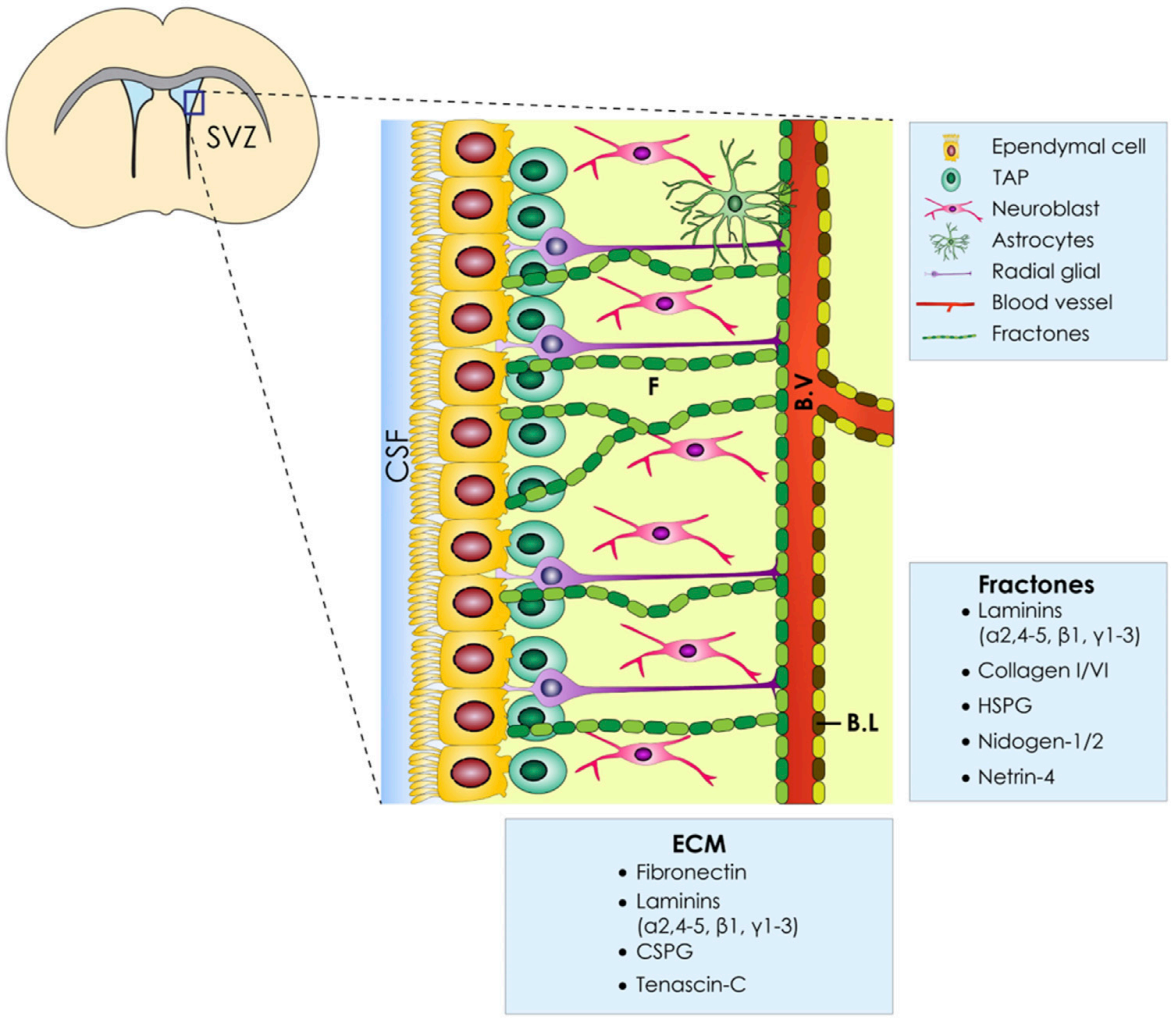
ECM of the ventricular–subventricular zone (V-SVZ) of the stem cell niche. Diagrammatic representation of the neural stem cell niche cellular constituents (E, ependymal Cells; NSC, neural stem cells; NB, neuroblasts; TAPs, transit amplifying progenitors; BV, blood vessels; ECM, extracellular matrix; BL, basal lamina). Moreover, the ECM of the SVZ has several components named fractones (F) which are in proximity with all cell types.

As it becomes obvious, a thorough understanding of the SVZ architecture, apart from the different cell populations that comprise this stem cell niche, is needed. The brain ECM, whose composition changes depending on the developmental stage (Bandtlow and Zimmermann, 2000), is composed of three main layers: 1) the basal lamina comprised of laminin, fibronectin, and heparan sulfate which encircles the blood vessels, 2) perineuronal nets composed of hyaluronate, proteoglycans, tenascin R, and link proteins that surround neuronal bodies, and 3) smaller neurons and a neural interstitial matrix (Murphy et al., 2017). Another feature of the V-SVZ is the presence of “fractones” which are thin, highly-branched ECM structures that emanate from the vascular basal lamina and are either scattered along the ependymal wall or arise in the center of the pinwheel structures. In addition to laminin, heparan sulfate, collagen IV, nidogen, and perlecan have also been defined as fractone constituents, as shown in Figure 1 (Morante-redolat et al., 2019). Apart from its supportive role, the brain ECM plays a crucial role in proper neural development, and alterations in its organization are involved in a variety of cortical malformations and other neurodevelopmental disorders [reviewed by Milošević et al. (2014)]. Last, the ECM glycosaminoglycans and proteoglycans have been shown to modulate neural stem cell behavior, as it was analytically reviewed by Shabani et al. (2021).

For proper SVZ niche reconstitution, apart from growth factors, several other factors are important such as cellular, chemical, mechanical, and environmental ones. A proper cellular arrangement for organotypic co-culture with spatial patterning inside a tissue-specific decellularized ECM (dECM) bioink could mimic the environmental conditions and cellular architecture. Moreover, regarding the chemical and mechanical properties, the dECM bioink could resemble both the chemical ques and the native tissue mechanical stiffness. Besides, from the 3D gel–provided stiffness and structural integrity, another important factor for ultimately mimicking the mechanical forces exerted on the SVZ ependymal surface is CSF flow. The CSF is not only acting as a buffer, but also its flow-generated shear stress provides some basic mechanical entrainment of ependymal cells which is vital for the SVZ niche autoregulation (Pellicciotta et al., 2020). To reproduce this, a 3D dynamic approach should be developed using the available microfluidic technologies. The goal of this mini-review is to provide a brief overview of different established approaches for the development of biofabricated organotypic co-culture platforms that recapitulate NSC niche tissue regions.

## Prior Models to Recapitulate Tissue-Specific Organotypic Cultures

### Static Transwell Co–Cultures

Great interest has been shown on the possible applications that neuronal culture models could ultimately provide, such as tissue regeneration, physiologic and pathophysiologic properties, and drug toxicity or permeabilization. Early attempts that were made to mimic the brain complexity were co-culture of astrocytes along with endothelial cells on treated membranes (Neuhaus et al., 1991; Gaillard et al., 2001). These efforts along with the different cell populations and interactions between them comprised the neural tissue of the blood–brain barrier (BBB). These platforms not only enabled scientists to study the physiologic properties of this specific region in terms of pharmacologic permeability but also proved to be valuable and validated tools for studying the properties of these tissues under pathophysiologic conditions. Several other attempts regarding the BBB have already been described and vary mainly on the choice of the co-cultured cell types (Nakagawa et al., 2009; Hatherell et al., 2011) as well as on the decision of the materials for membranes (Neuhaus et al., 1991; Gaillard et al., 2001; Ma et al., 2005) or coating. In most of these approaches, a common trend observed was the use of Transwell membranes (Nakagawa et al., 2009; Hatherell et al., 2011; Stone et al., 2019) due to the fact that they offer a variety of options regarding the different cell type populations and seeding localization (Li et al., 2006; Stone et al., 2019). Regarding the material of choice, there were several previous attempts which involved the development of novel hydrogels and decellularized region–specific materials to ultimately mimic the brain microenvironment composition, structure, and interactions. Ma et al., in 2004 had produced a membrane based on silicon nitride and showcased that the pore size along with the coating material prohibited the permeabilization of astrocyte bodies through the membranes but allowed their intermediate filaments to pass through the pores, thus resembling better the physiologic tissue (Ma et al., 2005). Transwell platforms have also been used successfully for developing reliable models of the air–liquid lung interface, which later can ultimately be studied and output data from cancer growth and therapeutics (Hassell et al., 2017) till high-throughput screenings for drugs against the SARS-Cov-2 virus (Mulay et al., 2020). Eventually, as we will describe in more detail in the next *Dynamic Transwell Co-Cultures section*, these platforms had been widely customized regarding the membrane materials and integrated with microfluidic chips to better facilitate the dynamic conditions that comprise the stem cell niches.

### Dynamic Transwell Co*-*Cultures

Another important factor that contributed substantially to the wide applicability of the Transwell setup is the fact that it can be used both for static and dynamic studies. The integration of microfluidic chips with membranes allows researchers to produce specific regions on a chip and study the complex interactions in an *in vitro* dynamic model. Shin et al. had produced a 3D micro Transwell device with two distinct flows of culture media through the microfluidic channels (Shin et al., 2014) in order to produce a vascularized 3D model of the NSC niche. They showcased that the spatiotemporal properties of the produced NSCs were found to influence the differentiation capacity of astrocytes. Regarding the development of 3D dynamic BBB model chips, Herland et al. utilized a collagen-based gel with embedded cells within a microfluidic channel to study the human analogue of an inflammatory response (Herland et al., 2016). Interestingly, they found that the dynamic model is more physiologically relevant compared to a static Transwell culture, regarding the release of cytokines produced by an inflammatory stimulation. In addition, Wang et al. successfully established a microfluidic array for constructing NSC *in vitro* niches and thus studied the cell fate decisions in various culture conditions, such as the perfusion rate and the material of choice (Wang et al., 2017). In the same year and in a similar manner, Hassell et al. demonstrated that human lung cancer cells can grow within an organ-on-a-chip culture device that mimics the dynamic lung structure and function (Hassell et al., 2017), pointing out the versatile usage of these platforms. Consequently, researchers are currently trying to miniaturize the scale and the processes in 96-well air–liquid cultures (Bluhmki et al., 2020) in an effort to produce high throughput, reproducible, and reliable setups by reducing the space and the amount of expensive consumables. Thus, developed customizable devices and platforms proved to be promising tools for studying tissue regeneration, physiologic and pathophysiologic properties, and drug screening analysis regarding the toxicity or permeabilization of drugs. Many other studies had already been conducted and are analytically described in the review articles (MofazzalJahromi et al., 2019; Oddo et al., 2019), utilizing similarly the Transwell setups along with microfluidic technologies.

## Biofabrication Advances in Tissue Reconstitution

The field of biofabrication allowed new research approaches to be involved and provided novel insights on how to reconstitute different tissues by incorporating different cell populations, embedded in supportive materials at precise regions to better mimic the physiologic architecture. Below, we provide a brief discussion on recent developments in the biofabrication field, which may prove to be suitable for adaptation and integration with dynamic systems mentioned above to have much more reliable 3D organotypic models. We will focus mainly on the materials and the methods that were utilized.

### Hydrogels and Decellularized ECM as Bioinks

Regarding the material of choice used in the biofabrication field, there are several previous demonstrations which involved the development of novel hydrogels and decellularized region–specific materials to ultimately mimic the brain microenvironment composition, structure, and interactions. First, hydrogels have been utilized to produce suitable microenvironments that not only promote cell adhesion and stemness maintenance but also support the mechanical and rheologic properties of the reconstituted tissue. Han et al., used chitosan and gelatin-based hydrogels to establish an NSC/ependymal cell co-culture system and showcased that gelatin promoted angiogenesis in this application and that their hydrogels can be injectable (Han et al., 2019). In a similar manner, a previous study generated from our laboratory utilized a mixture of alginate and gelatin-based bioink, in order to biofabricate an early 3D model of the SVZ niche (Ioannidis et al., 2020) using a custom-made 3D bioprinter. GelMA, a hydrogel consisting of gelatin methacryloyl with UV crosslinking capability, is also utilized in many research articles for the development of NSC niches with biofabrication approaches. For example, Li et al. used GelMA supplemented with laminin and alginate with embedded neurospheroids and bioprinted columns inside a supporting crosslinking bath containing embedded astrocytes to fabricate a model of the SVZ (Li et al., 2020). Other thermoresponsive hydrogels have also been used for the study of NSC behavior in 3D environments. Hsieh et al. (2015) developed a hydrogel consisting of biodegradable polyurethane and showed the impact of stiffness on NSC proliferation and differentiation. Moreover, effort has been put on developing new synthetic hydrogels that possess protein motifs in their network. These hydrogels can be tailored for a specific application as needed. Farrukh et al. investigated a polylysine (PL) hydrogel matrix and a 19-mer peptide containing the laminin motif IKVAV (IKVAV) on neuronal progenitor cells under different stiffness regimes (2 and 20 kPa) (Farrukh et al., 2017), whereas Balion et al. investigated synthetic hydrogel matrices of polyethylene glycol (PEG) functionalized with collagen-like peptide (CLP) alone or conjugated with either cell adhesion peptide RGD motif (mimicking fibronectin) or IKVAV motif (mimicking laminin) and their impact on cancer cell migration (Balion et al., 2020). Similarly, Aronsson et al. pointed out that a polypeptide-functionalized hyaluronan (HA) and polyethylene glycol (PEG)–based hydrogel possess a highly versatile engineering customization for biofabrication approaches (Aronsson et al., 2020). These hydrogels can be further processed for meeting the extrusion-based biofabrication standard criteria of injectability and shear thinning and even been processed to enhance the cellular attachment and cell–cell signaling in an effort to regulate or stimulate the stem cell behavior (Uman et al., 2020). Recent studies also show great interest in utilizing decellularized tissues to produce region-specific bioinks to be used in the biofabrication of microtissues. These dECMs can be further functionalized or be combined with other hydrogels, where their chemical manipulation is well-established to achieve better rheologic, crosslinking, and cell adherence properties. For instance, Mao et al. demonstrated the fabrication of liver microtissues using liver-specific dECM bioinks in combination with GelMA and utilized the digital light process-based bioprinting method (Mao et al., 2020). As for the NSC biofabrication approaches, Xu et al. used tissue-specific dECM hydrogels which were derived from the solubilized spinal cord and peripheral nerve tissue and showcased the differential material’s potential on enhancing and promoting cell proliferation and regeneration ability (Xu et al., 2021). Additionally, for a proper SVZ niche recapitulation, more research needs to be conducted to identify the specific ECM component ratios and ECM mechanical forces in order to develop a more precise tissue-specific bioink. The only parameter specified with certainty is CSF flow velocity, as Mestre et al. have demonstrated with particle-tracking studies in live mice that CSF flow was 18.7 µm/sec (Mestre et al., 2018). Finally, another important parameter for developing bioinks is the biofabrication approach. For instance, all those attempts mentioned above utilized injectable bioinks with different biomaterials (alginate, gelatin, GelMA, and dECM) depending on the utilized biofabrication technology. Thus, depending on the biofabrication approach, those materials need to be combined with laminin, fibronectin etc. with their specific ratios found on the native tissue, an issue that needs to be further exploited in the future.

### Living Bioinks

Apart from using dECMs, great effort has already been put in biofabricating microtissues using highly dense spheroids with various biofabricating approaches as previously analytically reviewed by Dalton et al. (2020). Briefly, Hall et al., established a living bioink consisting of highly dense callus organoids and were able to demonstrate the healing of critical-sized murine bone defects through spontaneous self-bio assembly of the microtissues (Nilsson Hall et al., 2020). In a similar manner and by utilizing extrusion and aspiration-assisted bioprinting, Daly et al. were able to fabricate a high-density microtissue model consisting of human mesenchymal stem cells which were able to fuse together during culturing and form larger constructs with precise deposition of cells (Daly et al., 2021). These living bioinks can be produced either by using a high number of spheroids fusing together in a scaffold-free manner or by being integrated within the biomaterials (Pedde et al., 2017).

### Current Limitations in Biofabrication

These methods have proven to be versatile for usage between different tissue’s reconstitution but lack an efficient way to mimic the dynamic nature of the physiologic parameters across different tissues. That is typically illustrated by the lack of the vascular network to efficiently provide the necessary nutrients for cell survival, which are required either when cultured for prolonged timepoints or when scaling up the size of the biofabricated constructs. To address this issue, Scott et al. developed both cardiac and cerebral microtissues fabricated from an organoid-based bioink, which contained different tissue-specific organoids and was afterward deposited in a sacrificial bath using the SWIFT method (Skylar-Scott et al., 2019). Their results suggest that the SWIFT biomanufacturing method was able to provide rapid perfusable constructs with increased cell viability of high-density microtissues and, therefore, paving the way for more accurate and sustainable prolonged culture approaches. Moreover, another important limitation in biofabrication is time consumption during the bioprinting process. Researchers are currently trying to develop photoresponsive bioinks combined with stereolithography-based bioprinting technologies to minimize the time needed for developing bigger constructs (Bernal et al., 2019). As mentioned above, all these attempts may not specifically involve the reconstitution of the brain, but they all point interesting novel methods that could be further developed and integrated with other established or non-established methods and platforms to fabricate the most relevant and dynamic organotypic 3D model of the SVZ stem cell niche. Static organotypic culture use is simpler to fabricate, cheaper to design, more readily available, and more broadly used. Also, there are many well-established protocols, but they fail to recapitulate the *in vivo* analog like the 3D static or dynamic biofabricated approaches do. Between 2D dynamic and 3D biofabricated organotypic cultures, the latter possess the advantage of specific deposition of cells, although the complexity and costs are highly increased. In our opinion, the most suitable model to recapitulate the SVZ analog is the 3D biofabricated dynamic model as it mimics the 3D ECM architecture, includes the different cell types comprising the SVZ, and recapitulates the dynamic flow rates of the CSF found in the SVZ native tissue. Their advantages and disadvantages are displayed in Table 1. Ultimately an *in vitro* model of the SVZ would be designed with spatial pattern– utilizing 3D dECM–based bioinks inside a microfluidic perfusion chamber. This will not only mimic the *in vivo* analog but will also miniaturize the samples and enable high-throughput drug screening analysis and discovery. Moreover, cells identified in biofabricated niches will further enhance the maturation process by further producing ECM proteins and by releasing chemical agents such as growth factors and cell signaling molecules. By accommodating all those factors, we strongly believe that this *in vitro* model will be closely be comparable to the *in vivo* analog. This SVZ *in vitro* model could also be utilized for the study of specific diseases whose pathophysiology is linked to the SVZ, such as CH, stroke, post-stroke SVZ neurogenesis (Cuartero et al., 2021), and SVZ-originating glioblastoma (Bardella et al., 2018).

**TABLE 1:** Summary of the different available protocols along with their advantages and disadvantages depending on their features and typical components.

Model type	Typical components	Niches reconstituted	Advantages	Disadvantages	Reference
Static OC	Transwells, coated membranes	Blood–brain barrier (BBB) lung niche cancer	Low complexity Low-cost available protocols	Lacks flow rates	Neuhaus et al. (1991), Gaillard et al. (2001), Ma et al. (2005), Li et al. (2006), Nakagawa S et al. (2009), Hatherell K et al. (2011), Hassell et al. (2017), Stone et al. (2019), Mulay et al. (2020)
Dynamic OC	Microfluidics, membranes, 3D cultures	BBB SVZ Lung niche Cancer	More relevant to physiology	High-cost specialized equipment	Shin et al. (2014), Herland et al. (2016), Wang et al. (2017), Mofazzal Jahromi et al. (2019), Oddo et al. (2019), Bluhmki et al. (2020)
Biofabricated-3D Bioprinted Static OC	3D cultures with hydrogels	BBB SVZ Bone	Better 3D microenvironment and precise deposition	High-complexity specialized equipment	Hsieh et al. (2015), Farrukh et al. (2017), Pedde et al. (2017), Mestre et al. (2018), Bernal et al. (2019), Han et al. (2019), Skylar-Scott et al. (2019), Aronsson et al. (2020), Balion et al. (2020), Cuartero et al. (2021), Dalton et al. (2020), Daly et al. (2021), Ioannidis et al. (2020), Li et al. (2020), Mao et al. (2020), Nilsson Hall et al. (2020), Uman et al. (2020), Xu et al. (2021)

## Conclusion

A niche is anatomically and functionally defined as a local tissue microenvironment capable of maintaining and regulating a particular kind of stem cell or progenitor (Morrison and Spradling, 2008). The abovementioned study highlights the ultimate need for the development of reliable *in vitro* tissue models that are capable of resembling adequately the physiology of the niche and the interactions occurring in this microenvironment. A variety of materials with various modifications have been investigated for the 3D culture of neural tissue, but even the most advanced materials struggle to mimic the complexity of the natural tissue. However, thanks to the utilization of organoid and hydrogel cultures, a favorable degree of complexity has been achieved compared to other *in vitro* methodologies. These cultures do, however, suffer from scale-up issues as they have poor nutrient diffusion abilities due to lack of vascularization. Even though 3D *in vitro* reconstitution of functional stem cell niche establishment still remains a big challenge, it becomes more and more evident that it holds great promise for its clinical significance in terms of disease modeling, pharmacologic applications, and surgical implantations in the future.
